# Flexible Bronchoscopy Under Bronchoscopist-Administered Moderate Sedation Versus General Anesthesia: A Comparative Study in Children

**DOI:** 10.1089/ped.2018.0887

**Published:** 2018-09-28

**Authors:** Pritish Mondal, Priti Dalal, Niruja Sathiyadevan, David M. Snyder, Satyanarayan Hegde

**Affiliations:** ^1^ Division of Pediatric Pulmonology, Department of Pediatrics, Penn State College of Medicine, Hershey, Pennsylvania.; ^2^ Division of Pediatric Anesthesia, Department of Anesthesia, Penn State College of Medicine, Hershey, Pennsylvania.; ^3^ Department of Anesthesia, Penn State College of Medicine, Hershey, Pennsylvania.; ^4^ Penn State College of Medicine, Hershey, Pennsylvania.; ^5^ Division of Pediatric Pulmonology, Department of Pediatrics, University of Chicago, Chicago, Illinois.

**Keywords:** bronchoscopy, children, bronchoalveolar lavage, pharmacotherapy, safety

## Abstract

***Background:*** Flexible bronchoscopy (FB) can be performed under bronchoscopist administered moderate sedation (BAMS) with a midazolam/fentanyl combination or general anesthesia (GA). However, the outcome of BAMS has not been well established in children. Currently, most of the centers prefer FB under GA. Both techniques have their advantages and disadvantages with implications for safety, complications, and diagnostic yield. The primary objective of our study was to evaluate the safety, time efficiency, and cost-effectiveness of FB under BAMS as compared with FB under GA in a similar setting.

***Methods:*** We performed a retrospective chart review to compare BAMS versus GA for FB in children. We recruited BAMS children (*n* = 295) from University of Florida (UF) Health Shands Children's Hospital, and GA children (*n* = 100) from Penn State Children's Hospital (PSHCH). Both the groups had similar indications, complexities, and procedural environments. Comparisons of various time-intervals including preprocedure time, sedation-induction time, scope time, and post-procedure time among different BAMS versus GA age-groups were the primary outcomes. The secondary outcomes were the determination of the rates of complications, the dosages of sedative/anesthetic, cost-effectiveness, and sedation patterns under BAMS.

***Results:*** FB under BAMS required significantly higher preprocedure times and sedation-induction times (*P* < 0.001** and *P* < 0.001** respectively) but shorter scope and post-procedure times compared with the GA group times (*P* < 0.001** and *P* < 0.001** respectively). Younger children had a deeper level of sedation for an extended period under BAMS. The costs for the sedation services and the complication rates were lower in the BAMS group compared with the GA group.

***Conclusion:*** Our study demonstrated the feasibility of BAMS in children. FB under BAMS had an advantage of lower cost and fewer procedural complications compared with FB under GA. Despite that, the safety of BAMS could not be conclusively established from this retrospective study. Moreover, BAMS can potentially compromise the diagnostic yield because the bronchoscopist is also responsible for monitoring sedation and managing the airway.

## Introduction

Flexible Bronchoscopy (FB) is an essential diagnostic tool in children and helpful in assessing the structure of the airways.^1^ In most children's hospitals, pediatric pulmonologists perform FB in an operating room (OR) setting with anesthesiologists providing the sedation.^2^ This setting, however, increases the expense of the procedure due to the high facility cost associated with use of the OR and charges for the anesthesiologist's time.^3^

FB in adult subjects has been well documented under various modes of sedation, including moderate sedation with midazolam and general anesthesia (GA) with propofol.^4^ However, similar data in the pediatric age group are lacking. Use of an opioid-benzodiazepine combination is a common strategy for procedural sedation in children.^5–7^ Anesthesiologists have also successfully utilized the combination of midazolam and fentanyl for moderate sedation and remifentanil and propofol for FB in children.^7,8^ Concurrent fentanyl has been used for Bronchoscopist-Administered Moderate Sedation (BAMS) since premedication with opioids helps to reduce the time to onset of sedation with midazolam.^9^ However, the efficacy of fentanyl in pediatric FB has not been extensively studied.^10,11^

BAMS is often utilized in adult FB, where a thorough understanding of the levels of sedation and the pharmacology of the sedative medications are prerequisites for the bronchoscopist.^12^ Conscious sedation (where the patients are still able to follow verbal commands) with a combination of a benzodiazepine and an opioid is usually sufficient for BAMS. Additionally, using BAMS is likely to save the added cost for OR and anesthesia; Hassan and his colleagues cited a model demonstrating the economic benefit of endoscopist-administered sedation as compared with anesthesiologist-assisted sedation for colonoscopy procedures.^13^

In most children's hospitals, FB is performed either by a dedicated sedation team in a bronchoscopy suite or with an anesthesiologist in an OR.^14^ BAMS is not commonly used in the pediatric population, although it has been a standard practice FB at the University of Florida (UF) Health. On the other hand, anesthesiologist-led GA is used for FB in children at the Penn State Health Children's Hospital (PSHCH). Both of these institutions are tertiary centers. Since the safety and efficacy of BAMS have not been established in children, and there are no published data available comparing FB under BAMS versus GA in pediatric age group, most children's hospitals are reluctant to implement BAMS as described in the adult literature.

The American Academy of Pediatrics (AAP) has specific guidelines for FB under moderate sedation.^15^ AAP recommends that all personnel, including the bronchoscopists, should be trained in pediatric advanced life support (PALS), and the child should be monitored with continuous capnography. The American Society of Anesthesiologists (ASA) uses its classification for risk estimation based on the physical status of the child on a 1–5 scale and recommends an evaluation by the pediatric anesthesiologist before opting for BAMS, unsupervised by an anesthesiologist.^16^

Our study is designed to bridge the knowledge gap and compared the outcome and experience of all FB performed over a period of two years at UF and at PSHCH. The objectives of the study are (i) to estimate the time efficacy and complication rate of FB under BAMS compared with that of FB under GA in a similar setting, (ii) to calculate the dosages of fentanyl and midazolam utilized for BAMS, (iii) to evaluate the degree and pattern of sedation achieved under BAMS, and (iv) to estimate the cost-effectiveness of FB under BAMS compared with FB under GA.

## Materials and Methods

We extracted and analyzed retrospective data from pediatric bronchoscopies done at UF (2011–2013) with an IRB-approved protocol. A comparative analysis of FB under BAMS versus GA was required for a better understanding of the time efficacy and relative safety of BAMS. At PSHCH, pediatric bronchoscopies are done in the procedure room under GA with propofol and supervised by a fellowship- trained pediatric anesthesiologist. To match the complexity of the cases with the BAMS group, we excluded certain cases from the GA group including tracheostomized children, lung biopsies, FB in an OR setting, and combined procedures with pediatric gastroenterologist or otolaryngologist.

### BAMS group

At UF, 90%–95% of procedures were completed in the bronchoscopy suite under BAMS, while the rest of the procedures were performed in the OR.

#### Bronchoscopy suite

The pediatric and adult pulmonology teams shared the bronchoscopy suite, located within the pulmonary diagnostic center. There were two bronchoscopy beds in the suite, and one of them was dedicated to pediatric bronchoscopies. Pediatric patients were scheduled at 1-h intervals, with a maximum of four patients scheduled for a half-day session.

#### Personnel and equipment

A sedation nurse and a pulmonary technician assisted at every FB. The nurse had the appropriate level of training in PALS, administration of sedative medications, and monitoring the level of sedation. Most of the technicians had the expertise to prepare the scope, handle the specimens, and clean the scope after the procedure. The bronchoscopist was required to have completed an online training program to acquire the credentials to perform BAMS. After completion of the training program, the bronchoscopist was required to apply for the privilege of performing BAMS. The bronchoscopy suite had a resuscitation cart equipped with a laryngoscope, endotracheal tubes, and reversal agents (flumazenil and naloxone). At PSHCH resuscitation training was not mandatory for bronchoscopists, although a few of the pulmonologists were PALS-trained. However, all the anesthetists at PSHCH had Pediatric Anesthesia fellowship training.

#### Medications

A combination of intravenous (iv) midazolam and fentanyl was used for BAMS. An initial dose of 0.05–0.1 mg/kg of midazolam and 1–2 mcg/kg/dose of fentanyl were used for children 0–12 years, with a maximum permissible cumulative dosage of 6 mg midazolam and 50 mcg fentanyl.^17^ For older children (>12 years) and for patients weighing 40 kg or more, the adult doses of 2–3 mg midazolam and 25–50 mcg fentanyl were considered as a starting dose. Incremental doses of 0.5 mg midazolam and 25 mcg fentanyl were given every 2–3 minutes as necessary until the desired level of moderate sedation was achieved.

#### The procedure

For outpatient procedures, patients were requested to check in an hour in advance of their procedure time. After registration and triage, secured IV lines were placed by the nurses in the procedure room. Once the desired level of sedation was achieved, FB was started by the bronchoscopist. Repeated doses of midazolam and fentanyl, if necessary, were verbally ordered by the bronchoscopist during the procedure. After the procedure, the patients were observed in the recovery area. The patients were discharged home once predefined discharge criteria were met. Discharge typically occurred 2–3 h postprocedure. ProVation MD was used for procedure documentation.^18^

#### Sedation scale

The Riker Sedation-Agitation Scale (SAS) and Richmond Agitation–Sedation Scale (RASS) were used to monitor levels of sedation during BAMS.^19,20^ The RASS ranges from +4 to −5, while SAS ranges from +7 to +1. Reduced scores indicate deepening of sedation.

### GA group

With IRB approval, we extracted and analyzed data from 100 pediatric bronchoscopies performed at PSHCH between 2015 and 2017. One of the pulmonologists, who performed more than half of those bronchoscopies, also performed a significant number of bronchoscopies at UF during 2012–2013. FB was usually started through the nasal passageway, while, in considerable number of the cases, laryngeal mask airway was used in later part of the procedure. Unlike BAMS, no sedation scale was maintained under GA.

### Time intervals

Time intervals we used the following time intervals to estimate the utilization of time for the procedure.

#### Preprocedure time

Preprocedure time was the time interval between when the child was brought inside the bronchoscopy suite/procedure room and initiation of sedation. This time was utilized for placement of an IV line, communication with the bronchoscopist, examination of the patient, documentation, calculation of the dose of sedation, and preparation of sedatives by the nursing team.

#### Sedation-induction time

Sedation-induction time the time interval between initiation of sedation/GA and beginning FB is defined as the sedation-induction time.

#### Scope time

Scope time the time interval between the insertion and removal of the scope.

#### Post-procedure time

Post-procedure time is defined as the time interval between the removal of scope and the time the patient leaves the bronchoscopy suite.

### We extracted the collective data on BAMS including the indication of all the bronchoscopies done from 2011 to 2013

For the GA group, we extracted indications for individual patients. Data were analyzed using SPSS. We used a 2-sample *t*-test to compare between the BAMS and GA groups and results were expressed as mean ± standard deviation.

## Results

A total of 395 children were audited. Of these, 295 belonged to BAMS group and 100 to GA group. There were no significant differences in weight within same age-groups when BAMS children were compared to GA children (Table 1). Both BAMS and GA patients were age-wise stratified into four groups for subgroup analysis: group 1 (0–23 months), group 2 (24–71 months), group 3 (72–119 months), group 4 (120 months and older).

**Table 1.** Age-Wise Distribution of Participants Comparing Weights Between Bronchoscopist Administered Moderate Sedation and General Anesthesia Subjects Demonstrates No Significant Differences

*Age group*	*Variables*	*BAMS (*n* = 295)*	*GA (*n* = 100)*	P
0–2 years	Sample size (*n*)	83	30	N/A
	Weight (kg)	9.0 ± 2.6	8.2 ± 2.6	0.20
>2–6 years	Sample size (*n*)	91	24	N/A
	Weight (kg)	16.1 ± 4.0	17.7 ± 5.0	0.15
>6–10 years	Sample size (*n*)	42	23	N/A
	Weight (kg)	29.0 ± 10.6	28.4 ± 8.7	0.82
>10–18 years	Sample size (*n*)	79	23	N/A
	Weight (kg)	53.2 ± 17.8	53.7 ± 14.4	0.89

BAMS, bronchoscopist administered moderate sedation; GA, general anesthesia.

### Indication

**FIG. 1.**
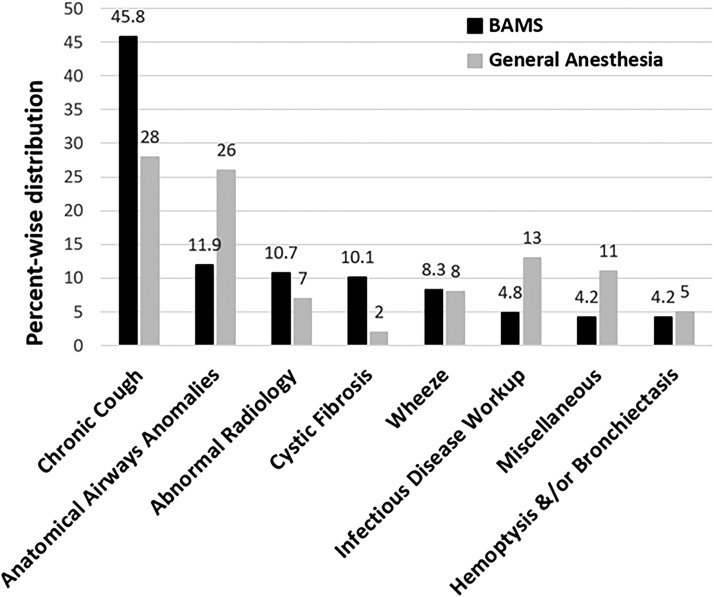
Indications for Flexible Bronchoscopy (percentage) under BAMS compared to General Anesthesia. Chronic cough and anatomical airway anomalies were among the most common indications in both of the groups. BAMS, bronchoscopist-administered moderate sedation.

Chronic cough was the most common indication for FB. followed by upper and lower airway anomalies including trachea-bronchomalacia in both BAMS and GA groups.^21^ 10.7% of the BAMS children had FB due to abnormal radiological findings, while 10.1% of GA children had FB as part of an infectious disease work up. The indications are demonstrated in Fig. 1.

### Complication

All BAMS were completed with spontaneous recovery without requiring reversal of sedation and without major complications or the requirement for an overnight stay in the hospital. Bronchoalveolar lavage was routinely performed as a component of FB. Two infants had reported complications, with one having desaturation to 69%, and the other having transient stridor. Only three toddlers had reported complications: a 1-year-old girl had bradycardia to 50/min that was resolved spontaneously, while another 2-year-old girl had desaturation to 80% requiring blow-by oxygen, and a 1-year-old girl had stridor noted at the end of the procedure that resolved upon awakening. A 4-year-old boy had mild stridor during FB, which responded to racemic epinephrine. Additionally, a 3-year-old boy had transient desaturation with epistaxis (<5 mL estimated blood loss) that was controlled with local compression/epinephrine drops.

For the GA group, all procedures were completed without major complications. However, 14 children had desaturation during FB; 10 of 14 were in group 1. Two patients had associated apnea, and 1 had a laryngospasm. A 3-month-old boy required intubation during the bronchoscopy due to upper airway obstruction. Out of 14 children with desaturation under GA, 2 children did not have proper documentation of SpO_2_ nadir. The average SpO_2_ nadir for the rest of the 12 patients was 85.6% ± 9.7%.

### Time intervals

The total time required for FB under BAMS (70.0 ±24.5 min) was significantly higher compared with FB under GA (51.2 ± 18.8 min) (*P* < 0.001**). We compared various time intervals between BAMS and GA subgroups (Table 2). We had different numbers of patients (N) corresponding to various BAMS time intervals depending on the documentation supplied by sedation nurses (Table 2). There was no difference among BAMS subgroups. In all age groups both preprocedure time and sedation-induction time were significantly higher in the BAMS group compared with the GA group, whereas scope time and post-procedure time were significantly lower in the BAMS group compared to the GA group (Table 2).

**Table 2.** Comparison of Time-Intervals Between BAMS and General Anesthesia Subjects According to the Age Groups

*Time intervals*	*Age groups*	*N (BAMS)*	*BAMS*	*N (GA)*	*GA*	P
Preprocedure time (min)	0–2 years	74	42.2 ± 19.3	30	13.8 ± 7.2	<0.001^**^
	>2–6 years	89	42.0 ± 22.4	24	10.5 ± 4.2	<0.001^**^
	>6–10 years	33	34.8 ± 19.8	23	12.5 ± 6.4	<0.001^**^
	>10–18 years	67	33.3 ± 15.2	23	9.5 ± 3.4	<0.001^**^
	All age-groups	263	40.0 ± 19.9	100	11.8 ± 5.8	<0.001^**^
Sedation-induction time (min)	0–2 years	67	8.6 ± 7.4	30	4.5 ± 3.9	<0.001^**^
	>2–6 years	72	12.5 ± 10.5	24	4.1 ± 4.7	<0.001^**^
	>6–10 years	25	10.6 ± 7.6	23	3.2 ± 1.7	<0.001^**^
	>10–18 years	50	9.5 ± 8.2	23	3.9 ± 3.5	<0.001^**^
	All age-groups	214	10.4 ± 8.9	100	4.0 ± 3.7	<0.001^**^
Scope time (min)	0–2 years	66	10.9 ± 4.8	30	16.3 ± 5.4	<0.001^**^
	>2–6 years	72	9.4 ± 4.7	24	17.3 ± 10.9	<0.001^**^
	>6–10 years	26	10.4 ± 4.9	23	12.3 ± 4.7	0.18
	>10–18 years	53	11.5 ± 5.9	23	14.5 ± 6.1	0.04^*^
	All age-groups	217	10.4 ± 5.1	100	15.2 ± 7.3	<0.001^**^
Post-procedure time (min)	0–2 years	25	9.2 ± 6.0	30	26.6 ± 13.3	<0.001^**^
	>2–6 years	39	8.6 ± 5.4	24	18.2 ± 9.0	<0.001^**^
	>6–10 years	10	6.0 ± 3.9	23	19.5 ± 12.4	<0.001^**^
	>10–18 years	22	9.9 ± 5.8	23	16.3 ± 8.7	0.006^*^
	All age-groups	96	8.8 ± 5.6	100	20.6 ± 11.8	<0.001^**^
Total time (min)	All age-groups	96	68.1 ± 24.2	100	43.6 ± 15.4	<0.001^**^

N represents the number of subjects with available data under each age-group category and varied according to the data availability. Data is presented as mean ± standard deviation. *P* value <0.05^*^ was considered significant and *P* value <0.001^**^ as highly significant.

### Sedation scale

**FIG. 2.**
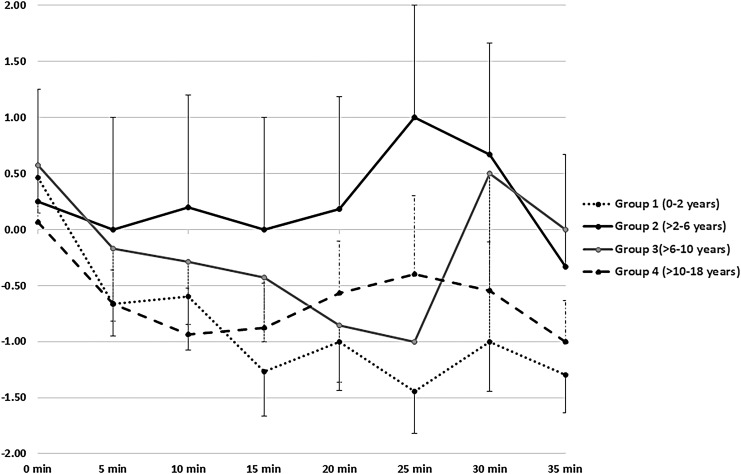
The pattern of sedation under BAMS in different age groups monitored using RASS for the first 35 minutes after administration of sedatives. RASS ranges from +4 to −5 and lower scores represent deepening of sedation. X and Y-axes represented the time-interval and RASS scores respectively. RASS, Richmond Agitation–Sedation Scale.

One hundred fifteen BAMS patients had sedation patterns monitored for 35 minutes, either using SAS or RASS. A deeper level of sedation was observed for an initial 15–20 minutes in most of the participants. Fifteen group-1 children, including 11 infants, were monitored using RASS. Likewise, SAS monitoring scores were available for 15 group-1 children, including six infants. Among all the participants monitored using RASS (Fig. 2), group-1 children had the deepest level of sedation for a longer period of time. An identical pattern was observed under the SAS, however, the increased depth of sedation was not so obvious in group 1.

### Medication

Within the BAMS group there were no statistical differences in per-kilo midazolam and fentanyl doses among the different age groups. The initial dose of midazolam was 0.05–0.1 mcg/kg. However, most of the children required a total dose of 0.10–0.15 mcg/kg of midazolam, which indicates the necessity of repeated administration (Table 3). Propofol was used for FB under GA.

**Table 3.** Dosage of Sedative/Anesthetics Used for Flexible Bronchoscopy Using Bronchoscopist Administered Moderate Sedation and General Anesthesia

*Age groups*	*Sedative/anesthetics*	*BAMS*	*GA*
0–2 years	Midazolam (mg/kg)	0.12 ± 0.05	N/A
	Fentanyl (mcg/kg)	1.43 ± 0.62	N/A
	Propofol (mg/kg)	N/A	8.4 ± 4.9
>2–6 years	Midazolam (mg/kg)	0.15 ± 0.06	N/A
	Fentanyl (mcg/kg)	1.62 ± 0.59	N/A
	Propofol (mg/kg)	N/A	5.6 ± 3.8
>6–10 years	Midazolam (mg/kg)	0.12 ± 0.05	N/A
	Fentanyl (mcg/kg)	1.45 ± 0.67	N/A
	Propofol (mg/kg)	N/A	6.0 ± 3.8
>10–18 years	Midazolam (mg/kg)	0.10 ± 0.05	N/A
	Fentanyl (mcg/kg)	1.14 ± 0.64	N/A
	Propofol (mg/kg)	N/A	7.2 ± 2.6

### Cost estimation

The cost of FB varies considerably between institutions. The Centers for Medicare and Medicaid Services (CMS) published the final rule in 2017 which specified the current procedural terminology (CPT) codes to be used for procedural sedation in children under various circumstances considering age group, duration of procedure, and participation of a second physician (anesthesiologist) for sedation service.^3^ The CPT code thus utilized for billing would determine the amount of Medicare/Medicaid reimbursement. We summarized the CMS recommendation for physician time-based billing in Table 4. We also estimated reimbursement for BAMS versus GA from the data available in the CMS website (Table 4).^22^ Under the new rule, projected physician costs for the sedation services will be at least 3–4 times higher for the GA group, compared with the BAMS group under similar circumstances.

**Table 4.** Correct Coding Combinations for Time of Moderate Sedation

*Total intra-service time*	*Patient age*	*Moderate sedation performed by bronchoscopist codes*	*Model reimbursement by medicare/medicaid*	*Moderate sedation performed by second provider codes*	*Model reimbursement by medicare/medicaid*
<10 min	Any age	Not reported separately	N/A	Not reported separately	N/A
15–22 min	<5 years	99151	$29.78	99155	$119.13
	≥ 5 years	99152	$15.24	99156	$90.48
23–37 min	<5 years	99151 + 99153	$45.58	99155 + 99157	$188.39
	≥5 years	99152 + 99153	$31.04	99156 + 99157	$159.74
38–52 min	<5 years	99151 + 99153 × 2	$61.38	99155 + 99157 × 2	$257.65
	≥5 years	99152 + 99153 × 2	$46.84	99156 + 99157 × 2	$229.00

Model reimbursement columns have been added to the existing table, which indicates maximum reimbursement for each current procedural terminology code/codes for a locality (MAC locality 0111205 was taken as a sample locality).

Source: The table has been reproduced from the article “Moderate Sedation Changes for Bronchoscopy in 2017” with permission from Elsevier^25^.

## Discussion

Our study is the first of its kind to demonstrate that FB can be performed under BAMS in children. Despite having minimal complication rates as indicated by this research, the safety of BAMS cannot be conclusively established from a retrospective study, and results should be utilized with caution. About 3% of FB procedures under BAMS were abandoned at UF due to inadequate sedation or failure to secure an IV line. This result was identical to the adult study reported by José et al., who demonstrated that 254 of 258 (98.4%) scheduled patients effectively completed FB under BAMS.^2^

There is an ongoing debate on safety and efficiency of BAMS. de Blic et al. described a prospective study evaluating complications of FB under conscious sedation in a cohort of 1,233 children; 3.4% of them experienced complications.^23^ FB under midazolam compared FB under placebo has shown better tolerance and fewer side effects like dyspnea, during the procedure.^24^ Ravenna et al. analyzed the safety of FB under midazolam in adult patients and reported desaturation in some cases within 10 minutes post administration of midazolam.^25^ However, we did not find significant desaturation during BAMS, and our study reproduced the results cited by de Blic et al.^23^

**FIG. 3.**
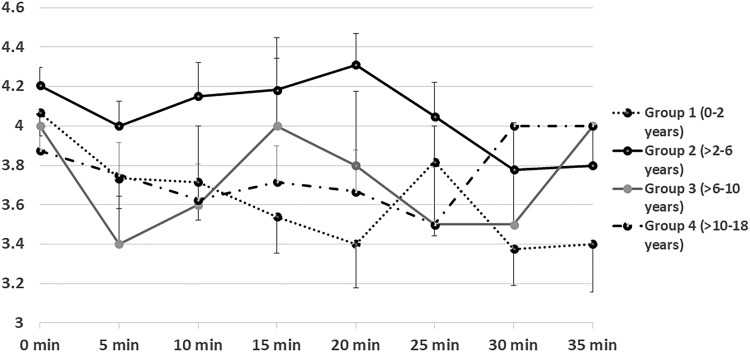
The pattern of sedation under BAMS in different age groups monitored using Riker SAS for the first 35 minutes after administration of sedatives. SAS ranges from +7 to +1 and lower scores represent deepening of sedation. X and Y-axes represented the time-interval and SAS scores respectively. SAS, Sedation-Agitation Scale.

Most of the children across all age groups maintained adequate depth of sedation for 20–25 min (Figs. 2 and 3) with the cumulative sedative dosage noted in the section on Medication. Since IV midazolam is short-acting, patients start to recover within 20–30 minutes after administration. This explains the decreased sedation after 25 minutes as demonstrated in the RASS data (Fig. 2). The doses of midazolam required for the successful completion of FB under BAMS were consistent across the age groups and well below the maximum recommended dose of 0.4 mg/kg.^9^ We found lower rates of complications under BAMS as compared with GA, especially in the younger children. The GA complication rate was a disadvantage and was likely due to the deeper level of sedation under propofol.

Differences in time intervals between the BAMS and GA groups can be explained based on the pharmacokinetics of the sedative agents and logistics of the procedures. Prolonged preprocedure time under BAMS was likely because the nurses utilized that time to place an IV in conscious children. In comparison, placing of an IV by anesthesiologists in a child already sedated with nitrous oxide was more time efficient. The average sedation-induction time under BAMS (8–13 min) was much longer compared with GA (3–4 min) because propofol has a faster onset of action and achieves the desired level of sedation more rapidly than does midazolam.^4,26^

It was surprising to see that the scope time was significantly less under BAMS as compared with GA. Since the bronchoscopist could concentrate only on the procedure, and the child had a deeper level of the sedation, we expected a shorter scope time under GA. However, it was possible that in the BAMS group, the bronchoscopists were in a rush to finish the procedure since the children used to resist considerably by removing the scope with their hand and vigorously moving their head. Moreover, the bronchoscopists were responsible for sedative dosage calculation and monitoring for adverse events. Those factors could have led to a sense of urgency that reduced scope time. If the hypotheses mentioned above are true, then one might argue against the effectiveness of BAMS and therefore its diagnostic yield. However, it was possible that reduced scope time in BAMS was multifactorial and that the presence of an anesthetist could have helped the bronchoscopist with monitoring of the sedation and airway management.

Unfortunately, comparing the diagnostic yield of FB under BAMS versus GA is beyond the scope of this study. High post-procedure time in the GA group was expected since GA entails a deeper level of unconsciousness based on the sedation continuum and hence leads to longer patient-waking times as compared to moderate sedation. Also, the children in the GA group were monitored by the anesthesiologist for a longer period of time to rule out an immediate postanesthetic complication. In-depth analysis of sedation scales was helpful for understanding the pattern of sedation under BAMS, which was necessary from a safety standpoint, especially in the absence of an anesthetist. Younger infants have immature hepatic microsomal oxidation systems, resulting in a slower metabolism of midazolam and a prolonged elimination half-life,^27^ and that could explain why the younger children had deeper levels of sedation for the longest durations.

The limitations of this study were its retrospective nature and selection of the GA group from a different institution (though with similar indications, complexities, procedural environments, and a bronchoscopist being common in both settings). At UF we performed 5%–10% of FB in the OR under GA, primarily for complex cases like tracheostomized children, patients with lung transplants requiring transbronchial biopsies, and for the procedures that also combined ENT or endoscopy. Therefore, the indications, time intervals, and procedure-related complications (like pulmonary hemorrhage, dyspnea, and desaturation) of FB under GA were very different compared with BAMS, and we did not have a matched group of GA patients for comparative analysis from UF. Therefore, we decided to recruit GA patients from a different health system that would match UF patients in regards to procedure logistics (procedure room versus OR), indications, and complexities of the cases.

Despite having an excellent safety profile, BAMS children at UF lacked adequate presedation screening for an ASA classification, which could have helped identify patients at risk. In spite of the moderate sensitivity of capnography, monitoring ETCO_2_ in intubated children by anesthetists provides an additional safety, which is not possible during BAMS.^28^ We noticed a difference in scope times in the two groups. Perhaps this difference could be attributed to the difference in experience of the bronchoscopists. Nonetheless, BAMS can potentially affect the diagnostic yield of FB since the bronchoscopist faces the additional tasks of monitoring sedation, managing airways, and performing FB in a semiconscious child.

Finally, it is worth mentioning that the latest ASA guideline, published in October 2017, recommends that the anesthesiologist should be actively involved in BAMS as an advisor and to oversee quality of care.^29^ However, our study indicates that, evern without oversight, BAMS can still be an option, especially considering its safety profile, lower cost, success in achieving the desired end-point with moderate doses of midazolam/fentanyl, lack of documented additional risks, and faster patient recovery times. Since serious adverse events during/following bronchoscopy are uncommon and the etiology of those events is often multifactorial, a larger sample size would be required to determine whether there are statistically significant differences in serious adverse event risks. Although sedation and resuscitation training are mandated for BAMS at UF, for those cases in which cardiopulmonary resuscitation is required, the presence of an experienced resuscitation team would help to achieve a better outcome.
